# Hydrophobic NADES-Derived Pumpkin Carotenoid Extract Attenuates Oxidative Stress and Mitochondrial Dysfunction in a Rat Model of Doxorubicin-Induced Cardiotoxicity

**DOI:** 10.3390/pharmaceutics18060662

**Published:** 2026-05-27

**Authors:** Milana Bosanac, Bojana Andrejić Višnjić, Aleksandra Popović, Marko Ljubković, Nikola Martić, Dejan Miljković, Alena Stupar, Biljana Cvetković

**Affiliations:** 1 Department of Histology and Embryology, Faculty of Medicine, University of Novi Sad, 21000 Novi Sad, Serbia; milana.bosanac@mf.uns.ac.rs (M.B.); dejan.miljkovic@mf.uns.ac.rs (D.M.); 2 Department of Physiology, Faculty of Medicine, University of Novi Sad, 21000 Novi Sad, Serbia; aleksandra.popovic@mf.uns.ac.rs; 3 Department of Physiology, University of Split School of Medicine, 21000 Split, Croatia; marko.ljubkovic@mefst.hr; 4 Department of Pharmacology, Toxicology and Clinical Pharmacology, Faculty of Medicine, University of Novi Sad, 21000 Novi Sad, Serbia; nikola.martic@mf.uns.ac.rs; 5 Institute for Pulmonary Diseases of Vojvodina, 21204 Sremska Kamenica, Serbia; 6 Institute of Food Technology, University of Novi Sad, 21000 Novi Sad, Serbia; alena.stupar@fins.uns.ac.rs (A.S.); biljana.cvetkovic@fins.uns.ac.rs (B.C.)

**Keywords:** doxorubicin, cardiotoxicity, carotenoids, NADES, oxidative stress, mitochondria, OXPHOS

## Abstract

**Background/Objectives:** Doxorubicin-induced cardiotoxicity (DIC) is driven by oxidative stress and impaired oxidative phosphorylation (OXPHOS). Antioxidant properties of carotenoids in vivo depend on extraction, while their direct role in mitigating DIC remains undetermined. This study evaluated the cardioprotective potential of natural deep eutectic solvents (NADES)-derived pumpkin carotenoid extract in a rat model of DIC. **Methods:** NADES-derived pumpkin pulp extract was characterized by spectrophotometry and HPLC-DAD. Wistar rats (*n* = 30) were assigned to six groups: C (control), N (NADES, 1 mL, p.o.), P (extract, 900 µg/kg body weight/day of total carotenoids, p.o.), D (doxorubicin, four i.p. doses, 2 mg/kg), ND (NADES/doxorubicin) and PD (900 µg/kg body weight/day of total carotenoids/doxorubicin). The activity of antioxidant enzymes and mitochondrial respiration in saponin-permeabilized left ventricular fibers using a Clark-type oxygen electrode) was measured. **Results:** Compared to control, cardiac antioxidant enzyme activities, mitochondrial respiration in complex I–linked respiration, ADP-supported OXPHOS, maximal respiratory capacity, and complex II and IV-linked respiration were significantly impaired by doxorubicin and unaltered by NADES. Co-administration of the extract significantly improved antioxidant enzyme activities (GSH-Px: D group 45 vs. PD group 95 nmol/mg proteins; GR: D group 95 vs. PD group 145 nmol/mg proteins; GST: D group 15 vs. PD group 22 nmol/mg proteins; SOD: D group 9 vs. PD group 17 U/mg proteins) and attenuated mitochondrial respiratory dysfunction compared with doxorubicin-treated group, indicating partial preservation of electron transport system capacity. **Conclusions:** Despite limitations of the study (single sex, single dose), results suggest NADES-based carotenoid extracts have cardioprotective properties in DIC by enhancing antioxidant defenses and supporting mitochondrial respiration.

## 1. Introduction

Doxorubicin (DOX) is a highly effective chemotherapeutic agent, but its clinical application is significantly constrained by treatment-related adverse effects. Among these, doxorubicin-induced cardiotoxicity (DIC) represents the most common long-term complication [1]. The clinical relevance of DIC has become increasingly evident due to the rising incidence of cancer, improved patient survival, and the expanded use of doxorubicin in individuals with pre-existing cardiovascular risk factors [2]. The pathogenesis of DIC is complex and involves multiple mechanisms, including oxidative stress, Topoisomerase II–mediated damage, mitochondrial dysfunction, and activation of regulated cell death pathways [3,4,5].

Mitochondria are highly dynamic organelles primarily responsible for producing ATP via oxidative phosphorylation (OXPHOS) [6,7]. Mitochondrial function is impaired by the direct interaction of DOX with Complexes I, II and IV proteins, causing damage to the electron transport chain (ETC). Additionally, doxorubicin leads to the inactivation of Complexes I, II and IV by increasing the production of reactive oxygen species (ROS), which, along with elevated calcium levels in the cytosol, heightens mitochondrial membrane permeability and results in the death of cardiomyocytes. On the other hand, the DOX-Topoisomerase IIβ complex can cause the inhibition of secondary OXPHOS. Because DOX preferentially accumulates in mitochondria, the high mitochondrial density in cardiomyocytes, together with relatively limited antioxidant capacity of the heart, may explain why cardiotoxicity represents the most frequent adverse effect of doxorubicin [4,7].

Every cell has a certain ability to neutralize ROS, thanks to antioxidant mechanisms, but when ROS production exceeds the capabilities of antioxidant defense, oxidative stress (OS) occurs [8]. Antioxidant defense can be enzymatic, forming the first line of defense (superoxide dismutase, catalase, glutathione peroxidase, glutathione S-transferase, glutathione reductase), or non-enzymatic, providing the secondary line of defense (glutathione, vitamins E and C, beta-carotene, polyphenols, thiol compounds, albumin, metallothionein, ubiquinone) [9].

Several compounds have been shown to exert cardioprotective effects against doxorubicin-induced cardiotoxicity, including coenzyme Q10, polyphenols, and other antioxidant agents that target oxidative stress and mitochondrial dysfunction [2]. These compounds primarily act by reducing ROS production, preserving mitochondrial integrity, and enhancing endogenous antioxidant defenses. It is believed that carotenoids share similar antioxidant properties, but due to their lipophilicity and ability to integrate into biological membranes, they may additionally stabilize mitochondrial structures and modulate electron transport chain function.

A wide range of pharmacological agents and dietary supplements have been investigated for the prevention and mitigation of doxorubicin-induced cardiotoxicity (DIC). More recently, growing attention has been directed toward the extraction and application of plant-derived bioactive compounds. Among these, carotenoids, the liposoluble plant pigments, are known to interfere with several central mechanisms of DIC formation. Their potent antioxidative, anti-inflammatory, and antithrombotic properties make them promising candidates for modulating coagulation disturbances and endothelial dysfunction associated with DIC. Notably, specific carotenoids such as β-carotene, lutein, and lycopene have been shown to attenuate oxidative stress and inhibit platelet aggregation, suggesting their potential therapeutic value in DIC prevention and management [10]. Building on this, pumpkin (*Cucurbita* spp.) represents an abundant and sustainable source of carotenoids, particularly β-carotene, lutein, and lycopene, which can contribute to antioxidant, anti-inflammatory, and antithrombotic effects [11].

However, despite the well-documented biological activities of carotenoids, their direct role in mitigating DIC remains insufficiently explored. Most studies have focused on individual compounds or conventional extracts, leaving a critical gap in understanding how carotenoid-rich matrices behave in physiological conditions, including their absorption, bioavailability, and systemic effects on the cardiovascular system under DIC-inducing stress [12]. In this context, Natural Deep Eutectic Solvents (NADES) have recently emerged as a green and efficient method to obtain carotenoid-rich extracts, preserving the chemical integrity and biological activity of lipophilic compounds while reducing reliance on toxic organic solvents [13,14]. NADES are generally considered promising green solvent systems because they can be prepared from naturally derived constituents and often exhibit low volatility and reduced flammability compared with conventional organic solvents. However, their biocompatibility, biodegradability, and toxicity cannot be generalized, as these properties strongly depend on the specific NADES composition, concentration, exposure route, and biological model and should be evaluated on a case-by-case basis, particularly when food, nutraceutical, pharmaceutical, or in vivo applications are considered [15,16]. Many NADES have demonstrated favorable biodegradability profiles compared to conventional organic solvents; however, this property is strongly dependent on their composition and should be evaluated on a case-by-case basis. A key advantage of NADES lies in their composition-dependent and tunable polarity, hydrogen-bonding capacity, and physicochemical properties, which enable efficient solubilization of a wide range of bioactive compounds, may improve the stability of labile constituents, and can reduce or eliminate the need for extensive downstream purification, thereby simplifying processing and lowering overall costs [15,17]. Accordingly, NADES can be tailored for the extraction of both hydrophilic and lipophilic compounds, depending on the selected hydrogen-bond donors/acceptors and the resulting polarity of the solvent system [18]. Hydrophobic NADES (e.g., menthol- or fatty acid-based systems) have shown suitability in the extraction of carotenoids and chlorophylls, whereas hydrophilic NADES (e.g., citric acid-based systems) are particularly effective for phenolic compounds, as demonstrated during systematic solvent screening of hemp sprouts. These findings highlight the importance of solvent customization for maximizing extraction yield and antioxidant potential [19]. Similarly, menthol-based NADES have enabled efficient extraction of astaxanthin from algal biomass at room temperature without additional energy input, while ultrasound-assisted NADES systems have achieved high yields of carotenoids and tocopherols from tomato processing by-products, with strong antioxidant activity. In addition to extraction efficiency, NADES have been reported to improve the bioaccessibility, shelf-life, and overall functional performance of plant-derived compounds [19,20,21]. Although sugar-based NADES may exhibit relatively high viscosity, hydrophobic fatty acid-based systems, such as octanoic acid and decanoic acid in a 3:1 molar ratio, offer reduced viscosity and improved mass transfer. These properties make them particularly suitable for extracting lipophilic carotenoids such as β-carotene, lutein, and lycopene from pumpkin matrices. When combined with ultrasound-assisted extraction (UAE), these systems further improve process efficiency, enabling higher yields, shorter extraction times, and the production of highly bioactive extracts [13,14].

While optimizing extraction parameters is essential to ensure maximal yield and preservation of bioactive integrity, the critical frontier lies in the in vivo evaluation of NADES-derived carotenoid-rich extracts. Such studies are indispensable for elucidating their bioavailability, pharmacokinetics, and systemic biological effects, providing the mechanistic and translational evidence necessary to bridge advanced green extraction technologies with real-world therapeutic applications. By directly linking sustainable extraction methods to physiologically relevant outcomes, this approach has the potential to accelerate the development of natural, effective strategies for mitigating complex conditions such as doxorubicin-induced cardiotoxicity.

Therefore, the primary aim of this study was to investigate the cardioprotective potential of carotenoid-rich extracts obtained from pumpkin in a model of doxorubicin-induced cardiotoxicity, through the assessment of oxidative stress and mitochondrial respiration parameters. In addition, the safety of NADES solvent in animals was examined through the parameters examined. To the best of our knowledge, this is the first study evaluating the effects of a NADES-derived pumpkin carotenoid extract on mitochondrial respiration and antioxidant defense parameters in a model of doxorubicin-induced cardiotoxicity.

## 2. Materials and Methods

### 2.1. NADES-Based Extraction and Analysis of Carotenoids from Cucurbita moschata

The plant material (*Cucurbita moschata*) was obtained from NS Seme (Novi Sad, Serbia) and cultivated at Rimski Šančevi. As the material was produced under controlled agricultural conditions and commercially available seeds were used, no voucher specimen was deposited. Lyophilized pumpkin (*Cucurbita moschata*) powder was used for extract preparation. The extraction protocol was based on methodology previously developed [14]. Hydrophobic NADES composed of caprylic acid (C8) and capric acid (C10) in a 3:1 molar ratio were selected to reduce viscosity and enhance extraction of lipophilic carotenoids. Previous studies, including the methodology reported by Stupar et al. [14], have demonstrated that fatty acid–based NADES improve carotenoid recovery, mass transfer, and antioxidant preservation compared to conventional solvents [13]. The total volume of NADES-derived pumpkin extract required for the study was calculated in advance and prepared in a single batch to ensure reproducibility and standardization across all experiments. Briefly, a NADES was prepared by mixing caprylic acid (C8) and capric acid (C10) in a 3:1 molar ratio and gently heating the mixture to 50 °C with constant stirring until a clear, homogeneous liquid formed. Pumpkin powder was extracted with NADES at a 1:20 (*w*/*v*) ratio using ultrasound-assisted extraction in an ultrasonic bath (TI-H 15, Elma Schmidbauer GmbH, Singen, Germany) at 45 °C, operating at a fixed frequency of 45 kHz, for 35 min. After extraction, the mixture was centrifuged at 5000× *g* for 10 min to remove insoluble residues, and the supernatant was filtered through Whatman No. 1 paper to yield a clear, carotenoid-rich extract. The extract was stored in amber vials at 4 °C, protected from light, until further analysis.

The total carotenoid content of the NADES-derived pumpkin extract was first assessed using spectrophotometric analysis, which indicated a concentration of 346.46 μg/mL. Absorbance was measured at 450 nm using a UV–Vis spectrophotometer, and total carotenoid concentration was calculated using the standard curve of β-carotene. Spectrophotometric measurements of the NADES-derived pumpkin extract were performed using the corresponding NADES solvent as the blank, following standard analytical practice for solvent-background correction. In addition to absorbance measurements at 450 nm, full UV–Vis spectra (350–550 nm) were recorded for both β-carotene standard and NADES-derived pumpkin extract. The extract exhibited characteristic carotenoid absorption maxima consistent with the reference compound, confirming compound identity. To obtain a precise and comprehensive profile of the individual carotenoids, the extract was further analyzed by high-performance liquid chromatography with diode-array detection (HPLC-DAD), following the methodology described by Matić et al. [11]. The carotenoid profile and quantification were determined using an Agilent 1200 HPLC system with a DAD detector and Zorbax SB C18 column (3.0 × 250 mm, 5 µm) (Agilent, Santa Clara, CA, USA). Separation was performed at 24 ± 1 °C with a flow rate of 1.5 mL/min using eluents (A) acetone/water (75:25, *v*/*v*) and (B) acetone/methanol (75:25, *v*/*v*) with a gradient from 0 to 100% B over 45 min. Extract samples were prepared by diluting 1 mL of the extract with 1 mL of isopropanol, followed by the addition of 1 mL of acetone. After mixing, 3 mL of mobile phase A (acetone/water, 75:25, *v*/*v*) was added to maintain consistency with chromatographic conditions. The resulting solution remained homogeneous, with no visible phase separation. The HPLC-DAD method was validated in terms of linearity, limit of detection (LOD), limit of quantification (LOQ), precision, and accuracy. Calibration curves showed excellent linearity (R^2^ > 0.999). LOD and LOQ values were in the low μg/mL range, while precision (RSD < 5%) and recovery (95–105%) confirmed the reliability and suitability of the method for carotenoid analysis. The chromatogram of the NADES solvent (blank) exhibited a stable flat baseline with no detectable peaks at the retention times corresponding to the target carotenoids, confirming the absence of matrix interference and demonstrating the selectivity of the analytical method.

**Figure 1:**
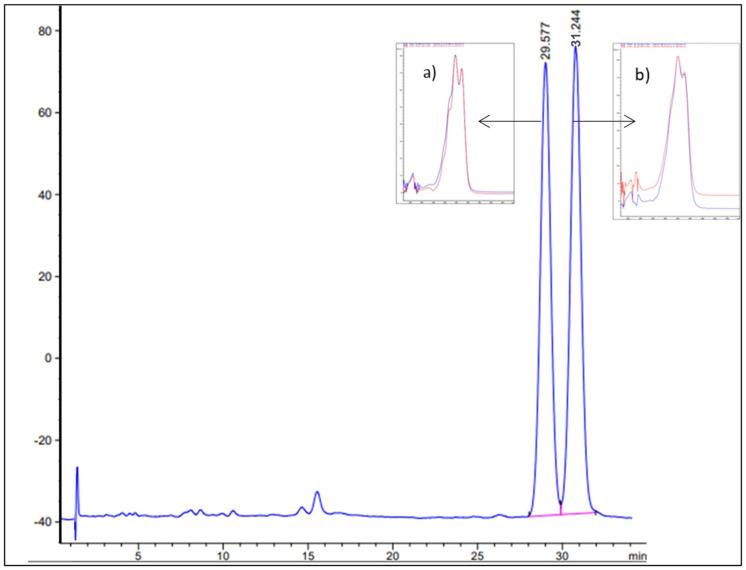
Representative HPLC-DAD chromatogram of the NADES-derived pumpkin extract. Major carotenoids identified include (**a**) α-carotene and (**b**) β-carotene (red line—standard; blue line tested sample). Peak identity was confirmed by both retention time matching and UV–Vis spectral comparison with reference standards. The overlaid UV–Vis spectra of the standards and corresponding peaks in the extract show high spectral similarity, confirming compound identity. No interfering signals from the NADES matrix were observed at the retention times of target analytes.

HPLC-DAD chromatographic analysis (Figure 1) clearly demonstrated efficient separation of carotenoids, with well-resolved and symmetrical peaks corresponding to α-carotene and β-carotene, indicating good chromatographic performance and method selectivity. HPLC-DAD analysis revealed that α-carotene and β-carotene were the predominant compounds, together representing more than 90% of the total carotenoid content, with concentrations of 147.91 μg/mL and 114.54 μg/mL (Figure 1).

### 2.2. Design of the Experiment

For the experiment, 30 healthy male Wistar rats were used. The animals were 3 months old, weighing from 250 to 300 g. We designed the study as a focused proof-of-concept investigation in a homogeneous male rat model, aiming to reduce biological variability, and therefore, only male animals were used.

During the experiment, animals were housed in the vivarium of the Department of Pharmacology, Toxicology, and Clinical Pharmacology at the Medical Faculty, University of Novi Sad, Serbia, under standard laboratory conditions. The animals were fed a standard diet with water available ad libitum. The Ministry of Agriculture, Forestry, and Water Management issued Decision No. 323-07-10388/2021-05 approving the implementation of the planned experiment on animals.

Animals were divided into 6 groups:

1. Negative control group (C) (*n* = 5)—1 mL saline, per os, for 20 days;
2. NADES solvent C8:C10 (N) (*n* = 5)—1 mL NADES solvent, per os, for 20 days;
3. Pumpkin pulp carotenoid extract (P) (*n* = 5)—900 µg/kg body weight/day of total carotenoids, expressed as β-carotene equivalents, per os for 20 days;
4. Positive control group–doxorubicin (D) (*n* = 5)—2 mg/kg of doxorubicin, intraperitoneally, on 8th, 12th, 16th and 20th day;
5. NADES solvent C8:C10 and doxorubicin (ND) (*n* = 5)—1 mL NADES solvent, per os, for 20 days and 2 mg/kg of doxorubicin, intraperitoneally, on 8th, 12th, 16th and 20th day;
6. Pumpkin pulp carotenoid extract and doxorubicin (PD) (*n* = 5)—900 µg/kg body weight/day of total carotenoids, expressed as β-carotene equivalents, per os for 20 days and 2 mg/kg of doxorubicin, intraperitoneally, on 8th, 12th, 16th and 20th day.

**Figure 2:**
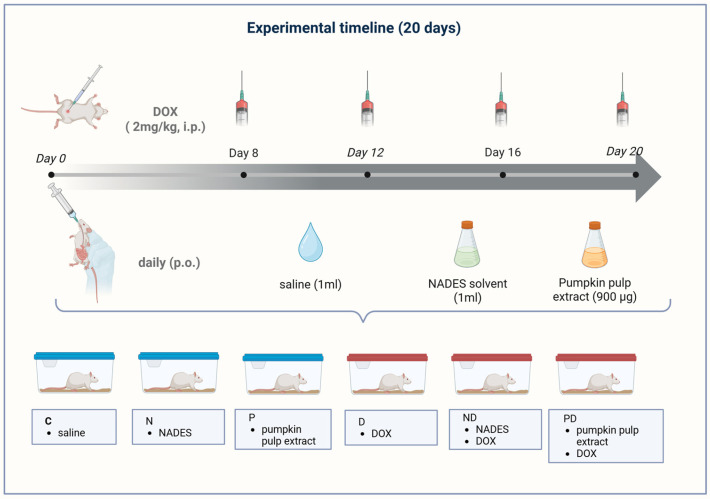
Experimental design. Created in BioRender. Andrejic Visnjic, B. (2026) https://BioRender.com/nvmv0at (accessed on 1 May 2026).

Per os administration meant treatment via an orogastric tube for small animals. On days when two substances were applied, the time period between two treatments was 4 h (Figure 2). Given that this manuscript is part of a larger experiment, the entire study design can be viewed in the paper [22].

### 2.3. Sacrifice of Animals

Animals were anesthetized on the 21st day using urethane (0.75 g/kg, intraperitoneally). After anesthesia, the chest of the animals was opened, after which cardiopuncture was performed. After deep anesthesia was confirmed, thoracotomy was performed, followed by cardiopuncture and excision of the beating heart. An autopsy was conducted on each animal, followed by collecting two sections of myocardial tissue—one for homogenization and analysis of oxidative stress parameters, and the other for examining mitochondrial respiration parameters.

### 2.4. Superoxide Dismutase, Glutathione Peroxidase, Glutathione Reductase and Glutathione S-Transferase Assays

Heart samples (1 g of heart tissue) were mixed with saline at a ratio of 1:4 *w*:*v*, creating a homogenate at 3 °C using an electric homogenizer, type B, Braun, Potter S (Melsungen, Germany). Then the samples were placed in an ultrasonic bath for 2 min, followed by centrifugation for 15 min at 1372 RCF (Relative Centrifugal Force), after which the supernatant and cytosol were separated [23,24].

The obtained supernatant was used for assays of superoxide dismutase (SOD), glutathione peroxidase (GPx), glutathione reductase (GR), and glutathione-S-transferase (GST). The activity of SOD was measured at 550 nm according to the method of McCord et al. [25]. The activity of glutathione peroxidase (GPx) was determined following the well-established methods [21], and the activity of glutathione reductase (GR) was determined using the methods of Glatzle et al. [25]. Glutathione S-transferase (GST) activity was measured using 1-chloro-2,4-dinitrobenzene as the substrate [24]. All measurements were obtained with a Boeco S-220 UV/Vis spectrophotometer (Hamburg, Germany).

### 2.5. Heart Isolation and Muscle Bundle Preparation

**Figure 3:**
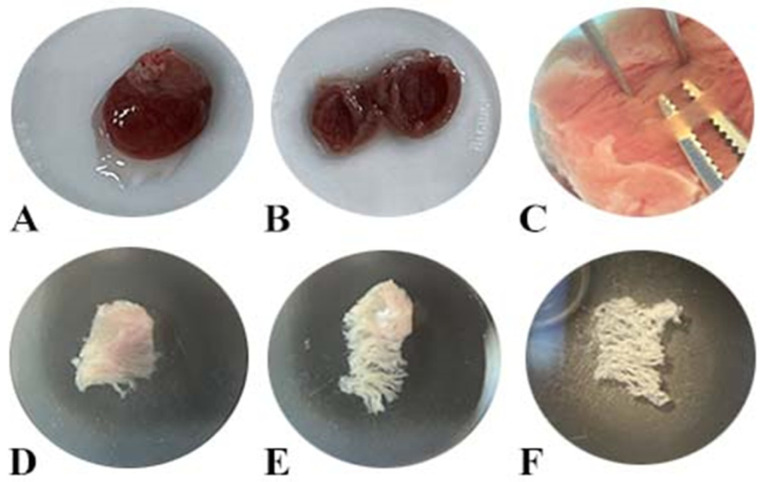
Preparation of myocardium muscle fibers: (**A**) The heart is placed in a Petri dish with ice-cold medium; (**B**,**C**) Sampling tissue pieces; (**D**) A pair of muscle bundles was cut from the myocardium of the left heart chamber; (**E**,**F**) Individual muscle fibers (obtained through careful mechanical dissection) were used for experiments.

The heart tissues were exteriorized via open-chest surgery and transported in an ice-cold medium consisting of 2.77 mM CaK_2_EGTA, 7.23 mM K_2_EGTA, 6.56 mM MgCl_2,_ 0.5 mM DTT, 50 mM K-methanesulfonic acid, 20 mM imidazole, 20 mM taurine, 5.77 mM Na_2_ATP and 15 mM phosphocreatine (pH 7.1) (Figure 3A). Further cutting of the rat heart and sampling tissue pieces was performed in the above medium at 2–4 °C (Figure 3B,C). A pair of muscle bundles (2–4 mm long, 1–1.5 mm in diameter, and about 5–7 mg of wet weight) was chopped with scissors from the myocardium of the left heart chamber (Figure 3D). Muscle bundles were sampled along the orientation of the muscle fibers to avoid mechanical damage to the cells themselves [25]. Careful mechanical dissection of individual muscle fibers was approached using extra-sharp antimagnetic forceps, leaving only small contact areas (Figure 3E,F). The dissection procedure was performed at 20× magnification (Boeco, Germany).

### 2.6. Preparation of Saponin-Skinned Muscle Fibers

The saponin treatment of dissected muscle fibers was carried out above a medium containing 50 µg/mL of saponin. The incubation lasted 30 min with gentle stirring at 4 °C to fully break down the sarcolemma [26,27]. Permeabilized muscle fibers were then washed in the MIR05 medium used for respiration measurements (0.5 mM EGTA, 3 mM MgCl_2_, 60 mM potassium-lactobionate, 20 mM taurine, 10 mM KH_2_PO_4_, 20 mM HEPES, 110 mM sucrose, 0.1% BSA, pH 7.1) by gentle stirring for 10 min at 4 °C. This washing process was repeated at least twice to ensure complete removal of saponin and other metabolites [28].

### 2.7. Measurement of Respiration

The Oxygraph plus apparatus (Hansatech Instruments, King’s Lynn, UK) was calibrated immediately before respiration measurements according to the manufacturer’s instructions. The oxygen consumption rate was measured using a Clark-type oxygen electrode in a water-jacketed glass chamber at 37 °C in 2 mL of a reaction MiR05 medium. Using Hamilton microsyringes (Hamilton, Reno, NV, USA), chemicals were added according to the experimental protocol as described previously [29] with some modifications: 10 mM pyruvate, 2 mM malate, 2.5 mM ADP, 10 mM glutamate, 0.5 μM rotenone (complex I inhibitor), 15 mM succinate, 2.5 μM antimycin A (complex III inhibitor), 0.5 mM TMPD (N,N,N′,N′-Tetramethyl-p-phenylenediamine) and 2 mM ascorbate. After the measurements, the samples were removed, and their wet weights were measured. The oxygen consumption rates were expressed as nmol O_2_/min/mg. The oxygen solubility in the medium was considered to be 230 nmol/mL.

### 2.8. Statistical Analysis

The software package SPSS 21 (Statistical Package for the Social Science 21) was used for statistical data processing. Numerical characteristics are presented using mean values (arithmetic mean, median) and measures of variability (value range, standard deviation, interquartile range), and attributive characteristics using frequencies and percentages. Data were tested for normality. For normally distributed data, one-way ANOVA followed by Tukey’s post hoc test was used. For non-normally distributed data, Kruskal–Wallis followed by Dunn’s multiple comparison test was used. For all statistical analyses, the probability limit of *p* < 0.05, i.e., *p* < 0.001, was taken as the criterion of statistical significance of differences.

## 3. Results

### 3.1. Analysis of Carotenoids from Cucurbita moschata

The approach used in this study enabled accurate separation, identification, and quantification of the carotenoids present in the extract. HPLC-DAD analysis revealed that α-carotene and β-carotene were the predominant compounds, together representing more than 90% of the total carotenoid content, with concentrations of 147.91 μg/mL and 114.54 μg/mL.

Cardiac oxidative stress and mitochondrial function were assessed to determine the protective potential of a NADES-based pumpkin carotenoid extract, as well as the tolerability of the corresponding NADES vehicle in a model of doxorubicin-induced cardiotoxicity. Analyses focused on key antioxidant enzymes and mitochondrial respiratory parameters, providing mechanistic insights into how carotenoids delivered via a sustainable NADES extract modulate redox homeostasis and bioenergetics function.

### 3.2. Dox Administration Altered Antioxidant Enzyme Activity

**Figure 4:**
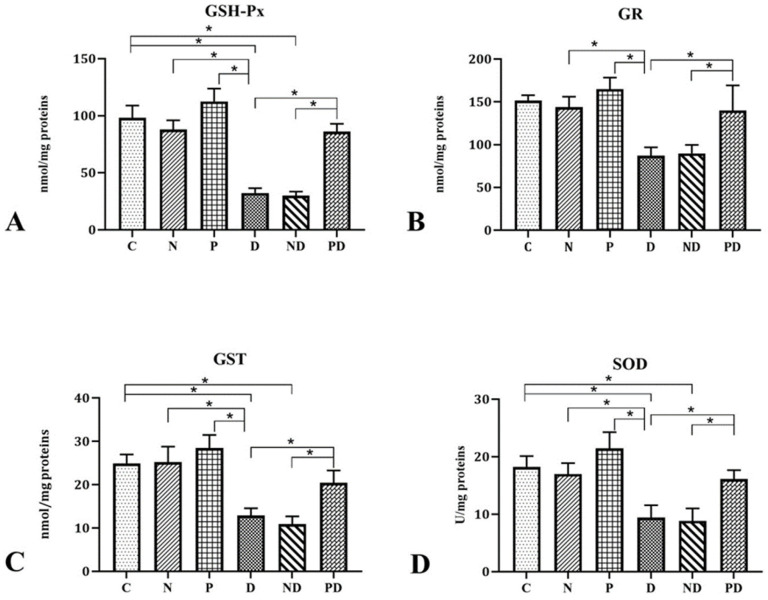
Antioxidative potential of pumpkin pulp carotenoid extract: (**A**) Glutathione peroxidase activity (GSH-Px; nmol/mg proteins, X ± SD); (**B**) Glutathione reductase activity (GR; nmol/mg proteins, X ± SD); (**C**) Glutathione-S-transferase activity (GST; nmol/mg proteins, X ± SD); (**D**) superoxide dismutase activity (SOD; U/mg proteins, X ± SD) in rat heart tissue. Each group consisted of 5 animals, while treatments include saline (**C**), NADES (C8:C10) solvent used for carotenoid extraction (N), pumpkin pulp carotenoid extract corresponding to 900 µg/kg total carotenoids (P), doxorubicin (**D**), co-treatment with NADES (C8:C10) solvent and doxorubicin (ND), and co-treatment of pumpkin pulp extract corresponding to 900 µg/kg total carotenoids and doxorubicin (PD). *—*p* < 0.001.

Four cardiotoxic doses of doxorubicin (group D) caused a statistically significant decrease in glutathione peroxidase enzyme (GSH-Px) activity (Figure 4A) compared to groups C, N, and P. Additionally, co-treatment with doxorubicin and NADES solvent (ND group) led to a significant reduction in GSH-Px activity compared to the control group. Treatment solely with NADES solvent (group N) and only with pumpkin pulp carotenoid extract corresponding to 900 µg/kg total carotenoids (group P) did not result in a significant change in enzyme activity compared to the group of animals given physiological solvent (group C). The application of the same pumpkin pulp carotenoid extract along with doxorubicin (group PD) significantly increased GSH-Px activity compared to groups D and ND.

In the group of animals treated with cardiotoxic doses of doxorubicin (group D), a statistically significant decrease in glutathione reductase (GR) activity (Figure 4B) was observed compared to groups N and P. In the same group (group D), there was a decrease in GR activity compared to the C group, although this was not statistically significant. Co-treatment with solvent and doxorubicin (ND group) caused a reduction in GR activity compared to groups C, N, and P; however, this change was not statistically significant. Application of NADES solvent (group N) and carotenoid extract from pumpkin pulp corresponding to 900 µg/kg total carotenoids (group P) did not result in statistically significant changes in enzyme activity compared to the C group. Treatment with pumpkin pulp carotenoid extract in animals also receiving doxorubicin (group PD) led to a statistically significant increase in GR activity compared to groups D and ND.

In animals that received doxorubicin (group D), a statistically significant decrease in glutathione-S-transferase (GST) enzyme activity (Figure 4C) was observed compared to groups C, N, and P. Co-treatment with NADES solvent and doxorubicin (ND group) caused a statistically significant rise in GST activity compared to the control group. Application of NADES solvent (group N) and carotenoid extract of pumpkin pulp corresponding to 900 µg/kg total carotenoids (group P) did not produce statistically significant differences compared to the control group. Treatment with pumpkin pulp carotenoid extract alongside doxorubicin (group PD) resulted in a statistically significant increase in GST activity compared to groups D and ND.

Administration of cardiotoxic doses of doxorubicin (group D) caused a statistically significant decrease in the activity of the enzyme superoxide dismutase (SOD) (Figure 4D) compared to groups C, N, and P. Similarly, the administration of doxorubicin in combination with NADES solvent (ND group) resulted in a statistically significant decrease in SOD activity compared to the control group. Application of NADES solvent (group N) and pumpkin pulp extract (group P) did not cause a statistically significant change in SOD activity compared to the control group. Treatment with pumpkin pulp carotenoid extract (group PD) caused a statistically significant increase in SOD activity compared to the group of animals that received only doxorubicin (group D) and co-treatment with doxorubicin and NADES solvent (group ND).

### 3.3. Effect of Dox Administration on OXPHOS Capacity

**Figure 5:**
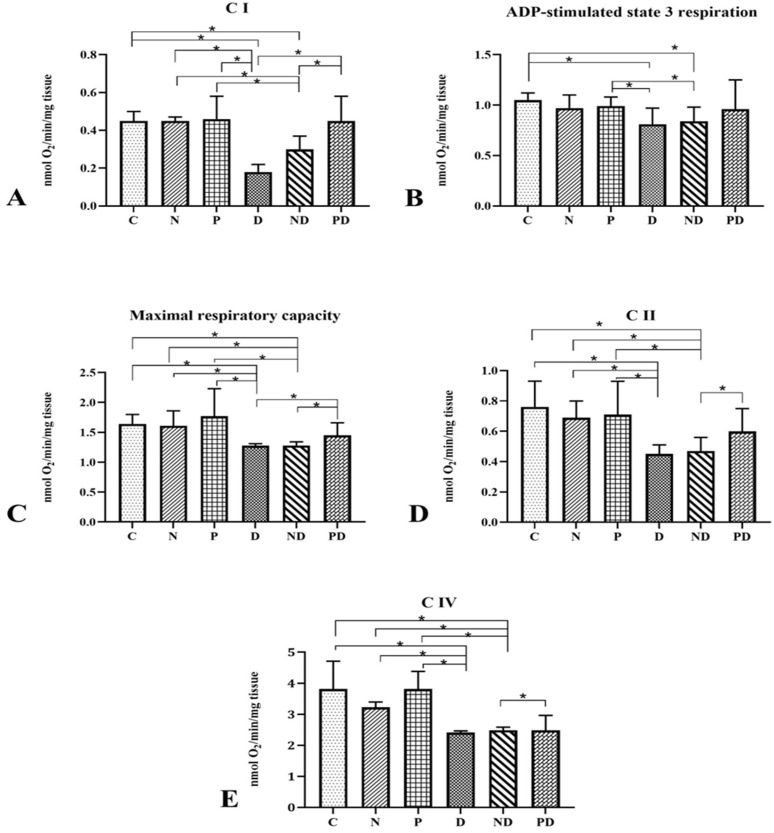
The protective role of pumpkin pulp carotenoid extract corresponding to 900 µg/kg total carotenoids in chronic cardiomyopathy induced by DOX and the effect of NADES solution on mitochondrial respiratory parameters. Oxygen consumption analysis was performed in saponin-permeabilized myocardium fibers of the left heart chamber. (**A**) Mitochondrial activities of complex I (CI) after the addition of pyruvate, malate, and glutamate as specific substrates, respectively. (**B**) Oxygen consumption rate under ADP phosphorylation conditions (state 3) in the presence of pyruvate, malate, glutamate, and ADP, respectively. (**C**) FCCP-stimulated maximal respiratory capacity in the presence of pyruvate, malate, glutamate, succinate, ADP, and FCCP, respectively. (**D**) Complex II-linked respiration after inhibition of Complex I with rotenone and addition of succinate. (**E**) Activity of complex IV (CIV) measured after inhibition of complex III with antimycin A and the addition of artificial substrates TMPD and ascorbate. Data are presented as mean ± SD; *n* = 5 animals per group. For mitochondrial respiration, one or two permeabilized fiber preparations were analyzed per animal, and animal means were used for statistical analysis. Data are presented as mean values  ±  SD of at least three independent experiments. Statistical significance was determined as * *p* < 0.001.

The oxygen consumption rates were measured in situ in saponin-permeabilized left ventricle fibers (Figure 5). After adding the substrates pyruvate, malate, and glutamate, the activity of Complex I (CI) was recorded (Figure 5A). The respiration rate range observed in the DOX group was significantly lower than in the N, P, ND, and control groups (*p* < 0.001). Also, co-treatment with DOX and NADES, as well as DOX and pumpkin pulp carotenoid extract (corresponding to 900 µg/kg total carotenoids), significantly inhibited CI activity (*p* < 0.001). At the same time, no difference was found between the group treated with NADES and the control (*p* > 0.05).

State 3 respiration was analyzed following the addition of pyruvate, malate, glutamate, and ADP (Figure 5B). The administration of cardiotoxic doses of doxorubicin caused a significant decrease in oxygen consumption rate compared to the values measured in groups N, P, ND, and the control (*p* < 0.001). Additionally, compared to the control, groups ND and PD showed a significant reduction in respiration rates (*p* < 0.001). In contrast, ADP-supported respiration in the group of animals treated with NADES was not significantly different from that measured in the control group (*p* > 0.05).

Maximal respiratory capacity (FCCP-stimulated) was measured in the presence of pyruvate, malate, glutamate, succinate, and ADP (Figure 5C). A decrease in oxygen consumption rate was observed in the group treated with DOX compared to N, P, ND, and the control (*p* < 0.001). Additionally, groups ND and PD showed reduced values compared to the control (*p* < 0.001). Conversely, the group of animals treated only with NADES exhibited no statistically significant difference in oxygen consumption rates compared to the control (*p* > 0.05).

Upon adding the complex I inhibitor (rotenone) and then applying substrate succinate, the activity of complex II was measured (Figure 5D). The rate of mitochondrial oxygen consumption in animals treated with cardiotoxic doses of doxorubicin was lower compared to untreated animals, as well as in groups treated with NADES and pumpkin pulp carotenoid extract corresponding to 900 µg/kg total carotenoids (*p* < 0.001). The mitochondrial respiratory efficiency in groups PD and ND was also significantly reduced compared to the control (*p* < 0.001). Conversely, treatment with NADES and pumpkin pulp carotenoid extract did not significantly affect the activity of complex II (*p* > 0.05).

Figure 5E showed oxygen consumption rates after inhibiting complex III with antimycin A and adding artificial substrates of complex IV (TMPD and ascorbate). In groups exposed to NADES and pumpkin pulp carotenoid extract, oxygen uptake rates did not differ from control (*p* > 0.05). Conversely, the activity of complex IV decreased significantly after administering cardiotoxic doses of doxorubicin compared to N, P, ND, and control (*p* < 0.001). The ETS capacity was also notably reduced following doxorubicin co-treatment with NADES and pumpkin pulp carotenoid extract, corresponding to 900 µg/kg total carotenoids (*p* < 0.001).

## 4. Discussion

Many long-term cancer survivors eventually die from heart failure rather than the primary disease [30]. Doxorubicin-induced cardiotoxicity (DIC) is a life-threatening complication, highlighting the importance of research into preventive agents, particularly herbal preparations. For each investigated substance, assessing safety is crucial, especially for new substances, where safety is still debated [30]. Food rich in carotenoids, especially β-carotene, is known for its preventive effects on the development and progression of various diseases, including malignant and cardiovascular illnesses [31]. Since humans cannot synthesize carotenoids, dietary intake or supplementation is necessary. In typical Western dietary patterns, α- and β-carotene are among the most frequently consumed carotenoids, commonly present in vegetables such as carrots, pumpkin, and spinach, while lycopene is predominantly derived from tomatoes and watermelon. Owing to its rich content of bioactive compounds, particularly carotenoids, pumpkin is increasingly recognized as a valuable functional food with potential health-promoting properties. Nevertheless, the bioavailability of carotenoids from plant-based sources, including β-carotene, is often limited, generally ranging between 10% and 65%, largely due to the structural complexity of plant matrices, including carotenoid–protein interactions, dietary fiber, and rigid cell walls that hinder efficient digestion and absorption [22,32]. To overcome these limitations, carotenoids can be administered in the form of extracts, which enhances their bioavailability. Extracting carotenoids from pumpkin thus offers a practical approach to enhance bioavailability, taking advantage of its affordability and rich carotenoid content [11].

Before discussing the physiological effects of pumpkin pulp carotenoids in an animal model of DIC, it is important to consider the chosen animal model and its specificities regarding carotenoid absorption and metabolism. In animals, β-Carotene and carotenoids in general are not synthesized and carotenoid plasma levels depend on intestinal absorption, doses and species. Although gerbils, ferrets, and preruminant calves are regarded as more suitable models for investigating carotenoid absorption and metabolism, their high cost, limited availability, and demanding handling requirements restrict their routine use in experimental settings. In addition, these models are not widely validated for studying diseases epidemiologically associated with carotenoids. In contrast, mice and rats are extensively used in research on oncology, immune function, and vitamin A deficiency, despite their limited capacity to absorb carotenoids in their intact form at physiological doses. The possible exception for using the rat and mouse would be experiments using high doses or a non-oral way of administration [33]. However, the oily consistency of the extract precluded non-oral administration due to the risk of embolism with intravenous injection and necrosis, inflammation, or adhesions with intraperitoneal administration. Additionally, even if funding and housing were available, current regulations prevent the import of more suitable species. Considering these factors, rats were selected for the present study, believing that this particular formulation of extract and specific solvent will enable sufficient absorption, which will be reflected through beneficial effects on the prevention of cardiotoxicity.

The issue of the effect of animal sex on the metabolism of carotenoids in the extract influenced the study design. Literature data provide information that both doxorubicin-induced cardiotoxicity and carotenoid metabolism may show sex-dependent differences [34]. Experimental evidence indicates that females may respond differently to doxorubicin exposure, partly due to sex-hormone-related cardioprotective mechanisms and differences in mitochondrial adaptation [35]. In parallel, recent data suggest sex-specific differences in β-carotene metabolism, including differential regulation of BCO1/BCO2 and estrogen receptor-related pathways [36]. Therefore, restricting the present experiment to one sex was intended to provide a more homogeneous biological background for the initial evaluation of cardioprotective efficacy. We agree that this limits generalizability, and we have now explicitly acknowledged this as a limitation and highlighted that future studies should include both sexes and be designed to address sex-specific responses directly.

The enzymatic conversion of carotenoids into bioactive metabolites was not fully understood until the early 21st century, when β-carotene-15,15′-dioxygenase (BCO1) and β-carotene-9′,10′-oxygenase (BCO2) were identified [37,38,39]. In rodents, the initial step in the metabolism of pumpkin-derived carotenoids involves their enzymatic conversion into retinal and other vitamin A–active metabolites. This process is mediated by β-carotene oxygenases, primarily BCO1, which cleaves provitamin A carotenoids, and BCO2, which exhibits a broader substrate specificity and acts on both provitamin A and non–provitamin A carotenoids. Both enzymes are predominantly expressed in the intestinal tissue; however, they differ in their subcellular localization, with BCO1 residing in the cytosol and BCO2 associated with the inner mitochondrial membrane [40]. One might argue that the effects documented in this research could relate to vitamin A-active products of carotenoid metabolism, rather than carotenoids themselves, but regardless of the final effector, the supplementation of carotenoids from pumpkin pulp extract corresponding to 900 µg/kg total carotenoids is a prerequisite for any effect recorded in this research. The absorption of carotenoids is highly dependent on the type of solvent used for their extraction and delivery. Consequently, increasing attention has been directed toward next-generation “green” solvents, particularly natural deep eutectic solvents (NADES). In contrast to conventional organic solvents, NADES are considered non-toxic and more biocompatible [41].

The prepared and examined extract from *C. moschata* was dominated by α-carotene (147.91 µg/mL) and β-carotene (114.54 µg/mL), together accounting for more than 90% of the total carotenoids. Prior work on *Cucurbita maxima* using a similar NADES protocol reported lutein (1.88 µg/mL), β-carotene (70.22 µg/mL), and β-cryptoxanthin (69.77 µg/mL) [14]. Importantly, the stability of carotenoids in NADES has been previously confirmed. Stupar et al. [14] demonstrated that pumpkin carotenoids stored in NADES in the dark at 4 °C exhibited minimal degradation (2.2%) after one month and only a 7.3% reduction after six months, supporting their good stability under appropriate storage conditions. Furthermore, recent studies consistently report superior extraction efficiency of NADES compared to conventional organic solvents. Tanrıver et al. [42] reported that NADES composed of L-menthol:propionic acid yielded 11.528 µg β-carotene/g using mechanical-mixing assisted extraction (MMAE) and 8.966 µg β-carotene/g using homogenization-assisted extraction (HAE) from C. moschata, outperforming traditional solvent-based methods. Similarly, Sportiello et al. [43] demonstrated that hydrophobic NADES systems extracted up to 1.165 mg β-carotene/mL from pumpkin peels, markedly higher than yields achieved with hexane or ethanol. These approaches enhance carotenoid recovery while preventing oxidative degradation, a limitation often observed with hexane or ethanol extraction. Collectively, these outcomes emphasize that NADES-based extraction represents a sustainable and highly efficient strategy for producing stable carotenoid-rich extracts with preserved bioactivity, supporting its use as a next-generation alternative to conventional solvent systems.

However, despite the clear advantages in yield, stability, and sustainability, very few studies have systematically evaluated NADESs themselves. Most research focuses exclusively on the extracted bioactives, leaving a gap in understanding how the solvent may interact with biological systems, influence bioavailability, or affect safety profiles. In the context of in vivo applications, such as cardioprotection or antioxidant interventions, it is critical to establish that NADESs do not exert adverse effects or interfere with the activity of the extracted compounds. Addressing this gap is essential for translating NADES-based extracts into safe and effective therapeutic or functional food products. Supporting this, in vivo studies demonstrated that a sorbitol–lactic acid NADES (3:1, 30% water) and its *Glycyrrhiza* extract caused no acute toxicity or mortality in mice (LD_50_ > 20 g/kg), confirming their safety for food, cosmetic, and pharmaceutical applications [44]. In addition, pumpkin carotenoid extracts obtained with a hydrophobic NADES composed of octanoic and decanoic acid (3:1) have shown cardioprotective effects in preclinical models of doxorubicin-induced cardiotoxicity, highlighting their therapeutic potential [22]. Due to the presence of a large number of mitochondria, as much as 40% of the volume of cardiomyocytes, the heart is more susceptible to damage caused by oxidative stress compared to other organs [4]. Considering these data and the fact that oxidative stress is one of the leading mechanisms of DIC, it is not unusual that DIC is the most common side effect of doxorubicin. Disruption of the antioxidant defense mechanisms of the heart caused by doxorubicin was proven by the reduction in the activity of antioxidant enzymes, both in earlier studies [45] and was also confirmed in the present study.

Antioxidant enzymes are a key marker and a good indicator of oxidative damage, and the role of oxidative stress in DIC has already been confirmed and documented. Many studies discuss the impact of doxorubicin on reducing the activity of antioxidant enzymes [46,47,48,49,50,51,52], yet have explored strategies to prevent this damage and maintain antioxidant defenses. Additionally, other researchers have shown that doxorubicin significantly reduces SOD activity [50,53]. Likewise, studies demonstrate that doxorubicin treatment causes a significant decrease in glutathione peroxidase (GSH-Px) [52,54,55] as well as glutathione reductase (GR) [54]. Furthermore, doxorubicin decreases glutathione S-transferase (GST) activity [53,55,56]. Consistent with these findings, the present study revealed a significant reduction in the activities of SOD, GSH-Px, GR, and GST in the D and ND groups compared with the control group. Results presented in this study are consistent with those of Hazaveh et al., who demonstrated that pumpkin seed extract can increase SOD activity, while different researchers confirm that other carotenoids effectively normalize SOD activity [57]. These findings are supported by other studies showing that the application of various carotenoids (carotenoids found in pumpkin pulp as well as other carotenoids) normalized GSH-Px activity following doxorubicin treatment [58,59]. An earlier study [60] explained the increase in SOD activity by the ability of omega-3 fatty acids to activate the ROS-sensitive signaling pathway Nrf2 in the mitochondria, which influences the increase in antioxidant enzyme activity. The results of the second part of this study showed that pumpkin pulp extract corresponding to 900 µg/kg total carotenoids statistically significantly increased the expression of Nrf2 antibodies, which was reduced after the administration of doxorubicin alone or the combination of doxorubicin and NADES solvent. These results indicate that pumpkin pulp extract has a cardioprotective effect, among other things, by regulating the Nrf2 signaling pathway [22].

Cardioprotective strategies against doxorubicin-induced cardiotoxicity have predominantly focused on compounds targeting oxidative stress and mitochondrial dysfunction, including coenzyme Q10, polyphenols, and modulators of redox-sensitive signaling pathways [2,48,49,50,51,52,53,54,55,61]. While these agents primarily act through ROS scavenging and preservation of mitochondrial bioenergetics, the present findings suggest that carotenoids may exert comparable protective effects through a partially overlapping, yet distinct mechanism. Owing to their lipophilic nature, carotenoids can integrate into biological membranes, including mitochondrial membranes, where they may contribute to structural stabilization and modulation of electron transport chain activity [31]. This property may be particularly relevant in the context of doxorubicin-induced mitochondrial impairment, which represents a central event in the development of cardiotoxicity.

The present study was designed as an initial proof-of-concept investigation aimed at evaluating the cardioprotective potential of a NADES-derived pumpkin carotenoid extract under doxorubicin-induced stress conditions. Therefore, we decided to use one dose for this early-stage in vivo research, to reduce biological variability and enable clearer mechanistic interpretation before proceeding to more complex dose-optimization designs, although this is a limitation of the study. Therefore, it is important to say that dose–response studies provide valuable information regarding the pharmacological profile of tested compounds and further research in this direction is needed.

The investigation into doxorubicin-induced ferroptosis indicated that the Nrf2/glutathione peroxidase signaling pathway plays a key role in its occurrence. Similarly, a potentially protective substance could activate Nrf2, leading to increased enzyme activity and reduced ROS accumulation [61]. Additionally, carotenoids were observed to enhance the activity of glutathione reductase and glutathione-S-transferase; however, more research is needed to determine the precise mechanisms. Analyzing the activities of SOD, GSH-Px, GR, and GST after administration of doxorubicin revealed a significant decrease in their activity in groups D and ND compared to the control group. Treatment with the NADES solvent did not mitigate the harmful effects of doxorubicin, and the diminished enzyme activity observed in the ND group is attributable solely to doxorubicin-induced oxidative damage. In contrast, co-administration of the pumpkin pulp carotenoid extract corresponding to 900 µg/kg total carotenoids with doxorubicin (group PD) resulted in a marked improvement in antioxidant enzyme activities. The observed increases in SOD, GSH-Px, GR, and GST activities compared with the D and ND groups suggest that the carotenoid-rich extract effectively enhanced the endogenous antioxidant defense system and mitigated oxidative damage induced by doxorubicin. Administration of the extract alone (group P) did not induce statistically significant changes compared with the control, confirming that it did not disturb physiological redox balance. These results imply that the beneficial effects observed in the PD group can be attributed to the antioxidant and membrane-stabilizing potential of carotenoids.

Recent research has highlighted the direct impact of doxorubicin on mitochondrial function, especially on the activity of the mitochondrial electron transport chain (ETC). Doxorubicin causes mitochondrial dysfunction by increasing ROS production and decreasing mitochondrial membrane potential [62]. Additionally, studies have demonstrated that doxorubicin inhibits the activity of AMP-activated protein kinase (AMPK), which is associated with cardiac hypertrophy [63]. Notably, doxorubicin is known to bind to cardiolipin, a phospholipid in the mitochondrial inner membrane that activates components of the ETC. Together, they form a strong, inseparable complex that significantly increases ROS production, causes mitochondrial dysfunction, inactivates Complexes I, II, III, and IV of the ETC, and consequently triggers apoptosis [7,51,63,64].

In this study, the cardiotoxic regimen of doxorubicin significantly decreased the activity of complexes I, II, and IV in groups D and ND compared to control groups N and P. The absence of a significant difference between groups N and C suggests that the reduction in activity in the ND group is due to the harmful effects of doxorubicin. The mixture of carotenoids in the pumpkin pulp extract corresponding to 900 µg/kg total carotenoids used in this study synergistically produced several beneficial effects on the activity of the examined complexes. First, the pumpkin pulp carotenoid extract effectively countered the cardiotoxic effects of doxorubicin, as shown by a significant increase in the activity of complexes compared to group D. Additionally, the pumpkin pulp carotenoid extract not only mitigated doxorubicin’s harmful effects but also, when used alone, increased Complexes I, II, and IV activity compared to the control group. It has been previously shown that lycopene can enhance the function of Complex I [65]. Frangiamone M. et al. used pumpkin extract from C. maxima, which contained β-carotene (49.29 ± 3.78 μg/g fresh substance), lycopene (19.25 ± 2.69 μg/g fresh substance), lutein (37.12 ± 3.08 μg/g fresh substance), zeaxanthin (16.99 ± 3.08 μg/g fresh substance), antheraxanthin (4.34 ± 0.74 μg/g fresh substance), astaxanthin (2.54 ± 0.39 μg/g fresh substance), and violaxanthin (9.09 ± 0.99 μg/g fresh substance). The study demonstrated that the extract increases Complex I activity, which in turn stimulates mitochondrial biopotential [66]. Furthermore, Peiran Lu and colleagues have shown that carotenoids can activate AMPK, which then inhibits signaling pathways leading to mitophagy, offering a possible explanation for the observed effects [67].

In addition to the above, it was also demonstrated that doxorubicin inhibits ADP-stimulated respiration in the mitochondria of mice or rat hearts treated in vivo with cardiotoxic doses of doxorubicin. This indicates that doxorubicin causes a direct inhibitory effect on one or more components of the ETC [4,68]. In the present study, rats treated with four doses of doxorubicin (groups D and ND) showed a statistically significant decrease in the oxygen consumption rate after adding the substrates pyruvate, malate, glutamate, and ADP compared to the control group. The NADES (C8:C10) solvent used for carotenoid extraction did not affect the changes in ADP-stimulated respiration, leading to the conclusion that the decrease in the ND group results from the cardiotoxic effect of doxorubicin. Administration of the pumpkin pulp carotenoid extract corresponding to 900 µg/kg total carotenoids (group P) caused a significant increase in ADP-stimulated oxygen consumption compared to groups D and ND (*p* < 0.05), demonstrating a clear stimulatory effect on mitochondrial respiration. The same dose of pumpkin pulp carotenoid extract co-administered with doxorubicin (group PD) managed to increase the rate of oxygen consumption, resulting in an upward trend compared with D and ND, but not enough to produce a statistically significant difference compared to groups D and ND. Additionally, these effects were also observed when analyzing the oxygen consumption rate, which relates to the maximum capacity of the ETC.

## 5. Limitations of the Study

This study was designed as an initial proof-of-concept investigation aimed at evaluating the cardioprotective potential of a NADES-derived pumpkin carotenoid extract corresponding to 900 µg/kg total carotenoids under doxorubicin-induced oxidative stress conditions. Although the findings suggest that carotenoid-rich pumpkin extracts obtained using NADES may attenuate doxorubicin-induced cardiac injury, several limitations related to the study design should be considered.

The use of only male animals enabled a reduction in hormonal and metabolic variability; however, this represents an important limitation, as both doxorubicin-induced cardiotoxicity and carotenoid metabolism may exhibit sex-dependent differences. Therefore, future studies should include female animals in order to assess possible sex-specific responses.

In addition, only a single dose level of the carotenoid extract formulated in NADES corresponding to 900 µg/kg total carotenoids was evaluated, which prevented assessment of dose–response relationships and optimal therapeutic range. Since the biological effects of NADES-based formulations cannot be interpreted solely on the basis of carotenoid concentration, future investigations should include multiple dosing regimens, formal toxicological evaluation, and pharmacokinetic characterization in order to better define the efficacy and safety profile of these formulations.

Although no adverse effects of the NADES vehicle alone were observed under the applied experimental conditions, comprehensive toxicological assessment of orally administered NADES systems was beyond the scope of the present study and should be addressed in future investigations.

Finally, the absence of functional cardiac imaging and pharmacokinetic analyses limits the translational interpretation of the present findings and therefore requires cautious interpretation of the results.

## 6. Conclusions

Doxorubicin induces pronounced oxidative stress and mitochondrial dysfunction in cardiac tissue, which represent central mechanisms underlying its cardiotoxicity. In this study, a NADES-derived pumpkin pulp carotenoid extract corresponding to 900 µg/kg total carotenoids partially preserved cardiac antioxidant enzyme activities and improved key parameters of mitochondrial respiration in a rat model of doxorubicin-induced cardiotoxicity, suggesting attenuation of oxidative stress and mitochondrial dysfunction associated with doxorubicin-induced cardiotoxicity. The absence of significant changes in the measured cardiac oxidative stress and mitochondrial respiration parameters in the NADES-only group suggests no detectable cardiac effect under the present experimental conditions. However, this does not replace a comprehensive toxicological evaluation.

Nevertheless, the present findings should be interpreted within the limitations of the study design, including the use of only male animals and a single-dose regimen. Future studies should include female animals and multiple dosing protocols in order to evaluate sex-dependent responses and dose–effect relationships.

## Data Availability

The original contributions presented in this study are included in the article. Further inquiries can be directed to the corresponding author.

## References

[1] Radić J.P., Kolarov B.I.N., Stefanović M.Z., Bosanac M.M., Cvetković B.R., Janičić S.D., Dolamić B.E., Ćuk D.R., Andrejić-Višnjić B.M. (2022). Cardiotoxicity of Doxorubicin: Causes, Diagnosis, Consequences and Possibilities of Prevention. Hosp. Pharmacol..

[2] Hammo A.A., Althanoon Z.A., Ahmad A.A. (2022). The Protective Effect of Coenzyme Q10 against Doxorubicin-induced Nephrotoxicity in Albino Rats. Rev. Electron. Vet..

[3] Sharma S., Parashar M., Lal K., Naik M., Tanwar S.S. (2026). Doxorubicin-Induced Cardiotoxicity: Comprehensive Pathway Insights and Advanced Preclinical Therapeutics. J. Appl. Toxicol..

[4] Wallace K.B., Sardão V.A., Oliveira P.J. (2020). Mitochondrial Determinants of Doxorubicin-Induced Cardiomyopathy. Circ. Res..

[5] Christidi E., Brunham L.R. (2021). Regulated cell death pathways in doxorubicin-induced cardiotoxicity. Cell Death Dis..

[6] DeBalsi K.L., Hoff K.E., Copeland W.C. (2017). Role of the mitochondrial DNA replication machinery in mitochondrial DNA mutagenesis, aging and age-related diseases. Ageing Res. Rev..

[7] Wu B.B., Leung K.T., Poon E.N. (2022). Mitochondrial-Targeted Therapy for Doxorubicin-Induced Cardiotoxicity. Int. J. Mol. Sci..

[8] Chaudhary P., Janmeda P., Docea A.O., Yeskaliyeva B., Abdull Razis A.F., Modu B., Calina D., Sharifi-Rad J. (2023). Oxidative stress, free radicals and antioxidants: Potential crosstalk in the pathophysiology of human diseases. Front. Chem..

[9] Zhu R., Wang Y., Zhang L., Guo Q. (2012). Oxidative stress and liver disease. Hepatol. Res..

[10] Sumalla-Cano S., Eguren-García I., Lasarte-García Á., Prola T.A., Martínez-Díaz R., Elío I. (2024). Carotenoids Intake and Cardiovascular Prevention: A Systematic Review. Nutrients.

[11] Matić M., Stupar A., Pezo L., Đerić Ilić N., Mišan A., Teslić N., Pojić M., Mandić A. (2024). Eco-Friendly Extraction: A green approach to maximizing bioactive extraction from pumpkin (*Curcubita moschata* L.). Food Chem. X.

[12] Ye Y., Yang L., Leng M., Wang Q., Wu J., Wan W., Wang H., Li L., Peng Y., Chai S. (2023). Luteolin inhibits GPVI-mediated platelet activation, oxidative stress, and thrombosis. Front. Pharmacol..

[13] Mišan A., Nađpal J., Stupar A., Pojić M., Mandić A., Verpoorte R., Choi Y.H. (2020). The perspectives of natural deep eutectic solvents in agri-food sector. Crit. Rev. Food Sci. Nutr..

[14] Stupar A., Šeregelj V., Ribeiro B.D., Pezo L., Cvetanović A., Mišan A., Marrucho I. (2021). Recovery of β-carotene from pumpkin using switchable natural deep eutectic solvents. Ultrason. Sonochem..

[15] Cannavacciuolo C., Pagliari S., Frigerio J., Giustra C., Labra M., Campone L. (2023). Natural Deep Eutectic Solvents (NADESs) Combined with Sustainable Extraction Techniques: A Review of the Green Chemistry Approach in Food Analysis. Foods.

[16] Halder A.K., Cordeiro N. (2019). Probing the Environmental Toxicity of Deep Eutectic Solvents and Their Components: An In Silico Modeling Approach. ACS Sustain. Chem. Eng..

[17] Chevé-Kools E., Choi Y.H., Roullier C., Ruprich-Robert G., Grougnet R., Chapeland-Leclerc F., Hollmann F. (2025). Natural deep eutectic solvents (NaDES): Green solvents for pharmaceutical applications and beyond. Green. Chem..

[18] Florindo C., Branco L.C., Marrucho I.M. (2019). Quest for Green-Solvent Design: From Hydrophilic to Hydrophobic (Deep) Eutectic Solvents. ChemSusChem.

[19] Ilić N.Đ., Stupar A., Matić M., Đerić M., Mišan A., Pojić M., Teslić N., Brunet J.Č., Mandić A. (2025). Enhancing bioactive compound extraction from hemp sprouts: Synergistic effects of NADES and ultrasound. Ind. Crops Prod..

[20] Al Fuhaid L., Wellman G.B., Kharbatia N., Farinha A.S., Vrouwenvelder J.S., Verpoorte R., Choi Y.H., Witkamp G.-J., Lauersen K.J., Fortunato L. (2024). Green extraction of pigment from astaxanthin-producing algae using natural deep eutectic solvents. Algal Res..

[21] Badea G.I., Gatea F., Litescu-Filipescu S.C., Alecu A., Chira A., Damian C.M., Radu G.L. (2025). Optimization of Green Ultrasound-Assisted Extraction of Carotenoids and Tocopherol from Tomato Waste Using NADESs. Molecules.

[22] Bosanac M., Stupar A., Cvetković B., Miljković D., Čanković M., Andrejić Višnjić B. (2025). Potential of Pumpkin Pulp Carotenoid Extract in the Prevention of Doxorubicin-Induced Cardiotoxicity. Pharmaceutics.

[23] Teofilović B., Tomas A., Martić N., Stilinović N., Popović M., Čapo I. (2021). Antioxidant and hepatoprotective potential of sweet basil (*Ocimum basilicum* L.) extract in acetaminophen-induced hepatotoxicity in rats. J. Funct. Foods.

[24] Stilinović N., Čapo I., Vukmirović S., Rašković A., Tomas A., Popović M., Sabo A. (2020). Chemical composition, nutritional profile and in vivo antioxidant properties of the cultivated mushroom *Coprinus comatus*. R. Soc. Open Sci..

[25] McCord J.M., Fridovich I. (1969). Superoxide dismutase: An enzymic function for erythrocuprein (hemocuprein). J. Biol. Chem..

[26] Pesta D., Gnaiger E. (2012). High-resolution respirometry: OXPHOS protocols for human cells and permeabilized fibers from small biopsies of human muscle. Methods Mol. Biol..

[27] Kuznetsov A.V., Mayboroda O., Kunz D., Winkler K., Schubert W., Kunz W.S. (1998). Functional im-aging of mitochondria in saponin-permeabilized mice muscle fibers. J. Cell Biol..

[28] Kuznetsov A.V., Veksler V., Gellerich F.N., Saks V., Margreiter R., Kunz W.S. (2008). Analysis of mitochondrial function in situ in permeabilized muscle fibers, tissues and cells. Nat. Protoc..

[29] Popovic A., Drljaca J., Popovic M., Miljkovic D., Marinovic J., Ljubkovic M., Kladar N., Capo I. (2022). Mitochondrial Energy Metabolism in Baby Hamster Kidney (BHK-21/C13) Cells Treated with Karnozin EXTRA^®^. Int. J. Morphol..

[30] Abraham K., Dorward A.M., Hurst Q., Pitt S.J. (2023). 6 Does mitsugumin 23 play a role in doxorubicin-induced cardiotoxicity?. Heart.

[31] Augustynska D., Jemioła-Rzemińska M., Burda K., Strzałka K. (2015). Influence of polar and nonpolar carotenoids on structural and adhesive properties of model membranes. Chem. Biol. Interact..

[32] Rošul M., Đerić N., Mišan A., Pojić M., Šimurina O., Halimi C., Nowicki M., Cvetković B., Mandić A., Reboul E. (2022). Bioaccessibility and uptake by Caco-2 cells of carotenoids from cereal-based products enriched with butternut squash (*Cucurbita moschata* L.). Food Chem..

[33] Seo J.S., Burri B.J., Quan Z., Neidlinger T.R. (2005). Extraction and chromatography of carotenoids from pumpkin. J. Chromatogr. A.

[34] Moulin M., Piquereau J., Mateo P., Fortin D., Rucker-Martin C., Gressette M., Lefebvre F., Gresikova M., Solgadi A., Veksler V. (2015). Sexual dimorphism of doxorubicin-mediated cardiotoxicity: Potential role of energy metabolism remodeling. Circ. Heart Fail..

[35] Sobhy M.H., Ismail A., Abdel-Hamid M.S., Wagih M., Kamel M. (2024). 2-Methoxyestradiol ameliorates doxorubicin-induced cardiotoxicity by regulating the expression of GLUT4 and CPT-1B in female rats. Naunyn Schmiedebergs Arch. Pharmacol..

[36] Oh Y., Kim J., Park Y.J., Kim Y. (2025). Male-Specific Effects of β-Carotene Supplementation on Lipid Metabolism in the Liver and Gonadal Adipose Tissue of Healthy Mice. Molecules.

[37] Widjaja-Adhi M.A.K., Golczak M. (2020). The molecular aspects of absorption and metabolism of carotenoids and retinoids in vertebrates. Biochim. Biophys. Acta Mol. Cell Biol. Lipids.

[38] Raghuvanshi S., Reed V., Blaner W.S., Harrison E.H. (2015). Cellular localization of β-carotene 15,15′ oxygenase-1 (BCO1) and β-carotene 9′,10′ oxygenase-2 (BCO2) in rat liver and intestine. Arch. Biochem. Biophys..

[39] Takitani K., Zhu C.L., Inoue A., Tamai H. (2006). Molecular cloning of the rat beta-carotene 15,15′-monooxygenase gene and its regulation by retinoic acid. Eur. J. Nutr..

[40] Martínez A., Cantero J., Meléndez-Martínez A.J., Paulino M. (2022). A Computer Simulation Insight into the Formation of Apocarotenoids: Study of the Carotenoid Oxygenases BCO1 and BCO2 and Their Interaction with Putative Substrates. Molecules.

[41] Bairaktari M., Konstantopoulou S.M., Malisova O., Gioxari A., Stratakos A.C., Panoutsopoulos G.I., Argyri K. (2025). Natural Deep Eutectic Solvents for Agro-Industrial By-Product Valorization: Emerging Strategies for the Development of Functional Foods Targeting Diabetes. Appl. Sci..

[42] Tanrıver K., Bilgin M., Şahin Sevgili S., Toprakçı Yüksel İ., Kurtulbaş Şahin E. (2025). Extraction of Carotenoids from Pumpkin (*Cucurbita moschata*) and Spinach (*Spinacia oleracea*) Using Environmentally Friendly Deep Eutectic Solvents (DESs). AppliedChem.

[43] Sportiello L., Marchesi E., Tolve R., Favati F. (2025). Green Extraction of Carotenoids from Pumpkin By-Products Using Natural Hydrophobic Deep Eutectic Solvents: Preliminary Insights. Molecules.

[44] Shikova V.A., Pozharitskaya O.N., Flisyuk E.V., Ivkin D.Y., Borovikov D.N., Balabanova O.L., Shikov A.N. (2025). Safety of NADES Extract of Glycyrrhiza Roots After Topical Application and Peroral Administration to Mice. Molecules.

[45] Wang A.J., Zhang J., Xiao M., Wang S., Wang B.J., Guo Y., Tang Y., Gu J. (2021). Molecular mechanisms of doxorubicin-induced cardiotoxicity: Novel roles of sirtuin 1-mediated signaling pathways. Cell. Mol. Life Sci..

[46] Zhang Y.Y., Yi M., Huang Y.P. (2017). Oxymatrine Ameliorates Doxorubicin-Induced Cardiotoxicity in Rats. Cell Physiol. Biochem..

[47] Al-Amir H., Janabi A., Hadi N.R. (2023). Ameliorative effect of nebivolol in doxorubicin-induced cardiotoxicity. J. Med. Life.

[48] Kuzu M., Yıldırım S., Kandemir F.M., Küçükler S., Çağlayan C., Türk E., Dörtbudak M.B. (2019). Protective effect of morin on doxorubicin-induced hepatorenal toxicity in rats. Chem. Biol. Interact..

[49] Sheibani M., Nezamoleslami S., Faghir-Ghanesefat H., Emami A.H., Dehpour A.R. (2020). Cardioprotective effects of dapsone against doxorubicin-induced cardiotoxicity in rats. Cancer Chemother. Pharmacol..

[50] Shosha M.I., El-Ablack F.Z., Saad E.A. (2023). Glycine protects against doxorubicin-induced heart toxicity in mice. Amino Acids.

[51] Nagy A., Börzsei D., Hoffmann A., Török S., Veszelka M., Almási N., Varga C., Szabó R. (2024). A Comprehensive Overview on Chemotherapy-Induced Cardiotoxicity: Insights into the Underlying Inflammatory and Oxidative Mechanisms. Cardiovasc. Drugs Ther..

[52] Ahmed O.M., Galaly S.R., Raslan M., Mostafa M.A.M. (2020). Thyme oil and thymol counteract doxorubicin-induced nephrotoxicity and cardiotoxicity in Wistar rats by repressing oxidative stress and boosting antioxidant defenses. Biocell.

[53] Li H., Xia B., Chen W., Zhang Y., Gao X., Chinnathambi A., Alharbi S.A., Zhao Y. (2020). Nimbolide prevents myocardial damage by regulating cardiac biomarkers, antioxidant level, and apoptosis signaling against doxorubicin-induced cardiotoxicity in rats. J. Biochem. Mol. Toxicol..

[54] Li Z., Chinnathambi A., Alharbi S.A., Yin F. (2020). Plumbagin protects against myocardial damage by modulating cardiac biomarkers, antioxidants, and apoptosis signaling in doxorubicin-induced cardiotoxicity in rats. Environ. Toxicol..

[55] Li D., Liu X., Pi W., Zhang Y., Yu L., Xu C., Sun Z., Jiang J. (2022). Fisetin reduces doxorubicin-induced cardiomyopathy both in vivo and in vitro by inhibiting ferroptosis through activation of the SIRT1/Nrf2 signaling pathway. Front. Pharmacol..

[56] Saleh Ahmed A.S. (2022). Potential protective effect of catechin on doxorubicin-induced cardiotoxicity in adult male albino rats. Toxicol. Mech. Methods.

[57] Hazaveh M.A., Abedi B., Ahmadabad S.R. (2021). Effects of aerobic training and pumpkin seed extract consumption on the heart and aorta oxidative stress biomarkers: A case of rats exposed to arsenic. J. Cardio-Thorac. Med..

[58] Varela-López A., Battino M., Navarro-Hortal M.D., Giampieri F., Forbes-Hernández T.Y., Romero-Márquez J.M., Collado R., Quiles J.L. (2019). An update on the mechanisms related to cell death and toxicity of doxorubicin and the protective role of nutrients. Food Chem. Toxicol..

[59] Zhuang C., Yuan J., Du Y., Zeng J., Sun Y., Wu Y., Gao X.-H., Chen H.-D. (2022). Effects of Oral Carotenoids on Oxidative Stress: A Systematic Review and Meta-Analysis of Studies in the Recent 20 Years. Front. Nutr..

[60] El Amrousy D., El-Afify D., Khedr R., Ibrahim A.M. (2022). Omega 3 fatty acids can reduce early doxorubicin-induced cardiotoxicity in children with acute lymphoblastic leukemia. Pediatr. Blood Cancer.

[61] Zhao X., Tian Z., Sun M., Dong D. (2023). Nrf2: A dark horse in doxorubicin-induced cardiotoxicity. Cell Death Discov..

[62] Jeong S.H., Kim H.K., Song I.-S., Lee S.J., Ko K.S., Rhee B.D., Kim N., Mishchenko N.P., Fedoryev S.A., Stonik V.A. (2014). Echinochrome A protects mitochondrial function in cardiomyocytes against cardiotoxic drugs. Mar. Drugs.

[63] Rawat P.S., Jaiswal A., Khurana A., Bhatti J.S., Navik U. (2021). Doxorubicin-induced cardiotoxicity: An update on the molecular mechanism and novel therapeutic strategies for effective management. Biomed. Pharmacother..

[64] Mei S., Hong L., Cai X., Xiao B., Zhang P., Shao L. (2019). Oxidative stress injury in doxorubicin-induced cardiotoxicity. Toxicol. Lett..

[65] Davies K.J., Doroshow J.H. (1986). Redox cycling of anthracyclines by cardiac mitochondria. I. Anthracycline radical formation by NADH dehydrogenase. J. Biol. Chem..

[66] Frangiamone M., Cimbalo A., Font G., Alonso-Garrido M., Manyes L. (2021). In vitro exposure to pumpkin extract induces a protective transcriptomic profile in blood brain barrier electron transport chain. Rev. Toxicol..

[67] Peiran L., Siau Y.W., Lei W., Dingbo L. (2020). Carotenoid metabolism in mitochondrial function. Food Qual. Saf..

[68] Zhao D., Jiang X., Meng X., Liu D., Du Y., Zhao L., Jiang H. (2023). Low-Dose Radiation Reduces Doxorubicin-Induced Myocardial Injury Through Mitochondrial Pathways. Dose Response.

